# Severe and Progressive Cellulitis Caused by *Serratia marcescens* Following a Dog Scratch

**DOI:** 10.1177/2324709619832330

**Published:** 2019-04-01

**Authors:** Deeti J. Pithadia, Erena N. Weathers, Rhonda E. Colombo, Stephanie L. Baer

**Affiliations:** 1 Medical College of Georgia, Augusta University, Augusta, GA, USA; 2 Madigan Army Medical Center, Joint Base Lewis-McChord, WA, USA; 3 Charlie Norwood Veterans Affairs Medical Center, Augusta, GA, USA

**Keywords:** *Serratia marcescens*, cellulitis, dog scratch, skin infection, immunocompromised

## Abstract

Soft tissue infections occur in over 30% of patients with chemotherapy-induced neutropenia. Gram-positive bacterial infections predominate early in neutropenia, and likelihood of infection by resistant bacteria and fungi increases with prolonged neutropenia. Prior infections and exposures influence the risk of rare pathogens. A 55-year-old woman with chemotherapy-induced neutropenia was scratched on her forearm by a dog. She cleaned the wound with isopropanol and was treated empirically with amoxicillin-clavulanate. Over the next 4 days, she developed fever along with erythema, edema, and mild tenderness of the forearm without purulence or crepitus. She was hospitalized and received empiric treatment with intravenous vancomycin, piperacillin-tazobactam, tobramycin, and voriconazole. Despite therapy, her fevers persisted and the cellulitis progressed for over a week. After 10 days of hospitalization, her neutrophil count began to recover and a bulla developed at the wound site. Culture of the bullous fluid grew *Serratia marcescens*, and antibiotics were switched to cefepime based on susceptibility. She defervesced and showed substantial improvement of cellulitis within 48 hours and was discharged on oral ciprofloxacin. *Serratia marcescens* skin infections are rare, and this may be the first report of *Serratia* cellulitis associated with trauma from dog contact. This case highlights the need to consider unusual pathogens based on exposure history and immune status and to obtain cultures from fluid collections or tissue in cases of treatment-resistant soft tissue infections.

## Introduction

Skin and soft tissue infections comprise approximately 30% of infections in patients with chemotherapy-induced neutropenia.^1^Gram-positive and antibiotic-susceptible gram-negative bacterial infections predominate in patients who are neutropenic for less than 7 days. The risk of infection by resistant bacteria, yeasts, and molds increases with prolonged neutropenia.^2^ Gram-positive cutaneous infections generally begin as focal regions of tenderness progressing to cellulitis, while gram-negative skin infections have diverse presentations including cellulitis, erythematous maculopapular lesions, and nodules.^3^ Past infections and exposures influence the risk of rare pathogens causing infection in immunocompromised patients.

We report a case of severe and progressive cellulitis following a dog scratch on the forearm of a patient who was profoundly neutropenic following chemotherapy.

## Case

A 55-year-old Caucasian female receiving chemotherapy for primary central nervous system lymphoma was scratched by a dog on her right forearm. She cleaned the wound with isopropanol and presented to the emergency department at a community hospital. She was discharged on oral amoxicillin-clavulanate, which she took as prescribed. Three days later, she was seen in oncology clinic for scheduled follow-up and was noted to have erythema surrounding the scratch site. The wound contacted tap water in the shower, but she denied other environmental exposures. Over the next 24 hours, she developed a fever and increased erythema, edema, and tenderness around the scratch site. She was subsequently admitted to our medical center.

Her history was significant for primary central nervous system lymphoma diagnosed 6 months prior. She completed consolidation chemotherapy 3 days prior to the dog scratch; a right-sided chest port remained in place. She had no prior history of immunocompromised state. She had no prior skin infections, significant trauma, or surgeries to the affected arm. She smoked 1 pack daily for many years but quit following her cancer diagnosis. The dog was up-to-date on vaccinations. Her outpatient medications included prophylactic acyclovir, fluconazole, and trimethoprim/sulfamethoxazole per standard chemotherapy protocol.

On admission to our medical center, she denied purulence, drainage, crepitus, or significant pain at the scratch site. She denied chest pain, dyspnea, abdominal pain, nausea, vomiting, diarrhea, or dysuria. She had a fever of 39.3°C and tachycardia at 110 beats per minute. Her respiratory rate, blood pressure, and oxygen saturation were within normal limits. She was awake, alert, and fully oriented. Her cardiac, pulmonary, and abdominal examinations were unremarkable. There was no erythema, purulence, or tenderness around her right-sided chest port. A 10-cm, well-circumscribed patch of erythema with a 1-cm central, darkly pigmented crust was present on her right forearm. The erythema had expanded from its earlier size denoted by an outline drawn 3 days earlier. The region was warm and mildly tender. She had 2+ radial and brachial pulses bilaterally and 5/5 motor strength of the bilateral upper extremities. No pain on flexion or extension of elbows, wrists, or fingers. Sensation was unaffected throughout the right upper extremity. Her laboratory findings were significant for undetectable white blood cell count. Hemoglobin was 11.7 g/dL, hematocrit was 34.0%, and platelet count was 19 000/mm^3^.

**Figure 1.**
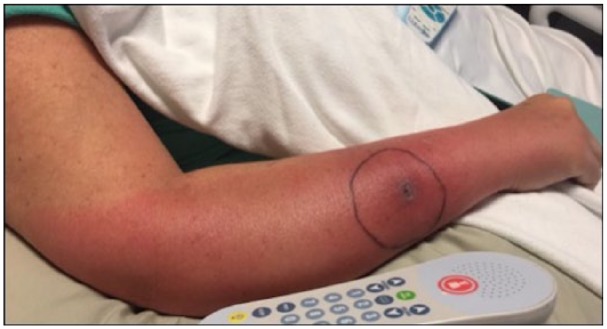
Appearance of patient’s arm on day 5 of hospitalization. The ulcerated region was the location of the dog scratch, and the drawn circle denoted the borders of erythema marked 3 days following the incident and 1 day prior to hospitalization. Borders of erythema were sharply demarcated, and edema was mild.

**Figure 2.**
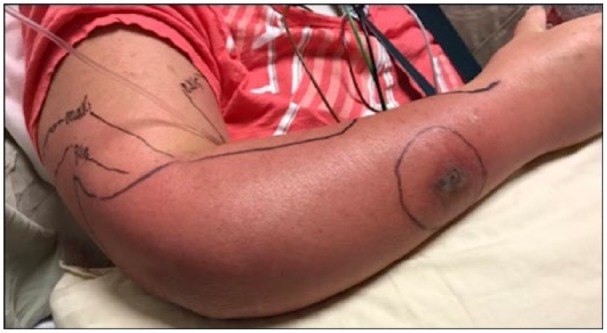
Appearance of patient’s arm on day 7 of hospitalization. Borders of erythema became less poorly demarcated and spread into the arm and medial forearm. The edema had extended into the hand and digits.

**Figure 3.**
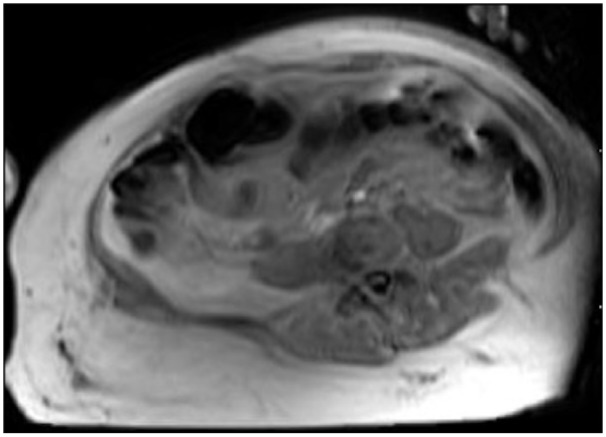
Magnetic resonance imaging study of right forearm on day 9 of hospitalization, ordered due to concern for compartment syndrome. The study demonstrated diffuse soft tissue cellulitis without abscess as well as myositis without pyomyositis.

Amoxicillin/clavulanate was discontinued, and vancomycin intravenous (IV) and piperacillin/tazobactam IV were begun at admission. Cultures of blood from her chest port and urine were found to grow *Pseudomonas aeruginosa* with identical susceptibilities, including to piperacillin/tazobactam and cefepime. Culture of blood taken at the same time from a peripheral site was negative, and all subsequent blood cultures during hospitalization were negative. Despite the empiric broad-spectrum antibiotic therapy, the cellulitis worsened. The borders of erythema became more sharply demarcated by hospital day 5 (Figure 1), and the patient experienced daily fevers with maximum temperature of 39.2°C. Clindamycin IV was added for its antitoxin effect on day 5. By hospital day 7, the erythematous patch became less well-circumscribed (Figure 2), and she developed increased pain and decreased strength (4/5) on flexion of the right wrist and digits. Ultrasound of the right arm showed soft tissue edema. Subsequent magnetic resonance imaging showed an elbow joint effusion and myositis without pyomyositis or abscess (Figure 3). Voriconazole IV was added on day 7 for empiric antifungal coverage. The patient’s chest port was removed on day 8 of hospitalization and was culture-negative.

On hospital day 10, the patient’s leukocyte count recovered to 3400/mm^3^ (neutrophils 2800/mm^3^, lymphocytes 400/mm^3^, monocytes 200/mm^3^) and a bulla developed adjacent to the site of the dog scratch. Serous fluid from the bulla was drained using sterile technique and grew *Serratia marcescens* on culture. Cefepime IV was initiated based on susceptibility results, and piperacillin/tazobactam and voriconazole were discontinued. She defervesced and experienced substantial improvement in the cellulitis over 48 hours. The patient was discharged home after 15 days of hospitalization on oral ciprofloxacin to complete a 14-day course of targeted antibiotic therapy. She was advised to keep her arm elevated to promote drainage of edema.

At follow-up, the patient continued to be afebrile after completing her antibiotic course. She received wound care at a community hospital. The wound healed completely within 2 months.

## Discussion

Cellulitis is infection of the dermis and subcutaneous fat most frequently caused by introduction of *Streptococcus* or *Staphylococcus* species into the skin through abrasions.^4^ Cellulitis secondary to traumatic contact, including scratches and bites, by domestic mammals is most commonly due to *Pasteurella multocida*.^5,6^ In patients with contaminated water exposure, *Aeromonas hydrophila, Vibrio vulnificus*, and *Pseudomonas aeruginosa* have been reported as culprits.^7,8^ *Pseudomonas aeruginosa* also is a common cause of skin infections and osteomyelitis in individuals suffering from puncture wounds.^9^

Due to the patient’s history of a penetrating wound with subsequent tap water contact in the setting of neutropenia, the eschar-like lesion on the arm resembling ecthyma gangrenosum, and the blood and urine culture results, *Pseudomonas aeruginosa* was initially believed to be the cause of cellulitis. Failure to respond to piperacillin/tazobactam, despite in vitro susceptibility, prompted further investigation. Although bullous cellulitis secondary to gram-negative septicemia is rare, *Pseudomonas aeruginosa*, which classically presents in the skin as ecthyma gangrenosum, has been documented as a cause in numerous case reports.^10^

*Serratia marcescens* is a gram-negative, motile, facultative anaerobic *Bacillus* that is pervasive in soil, water, and other damp environments. Prior to the mid-to-late 20th century, *Serratia marcescens* was not considered to be a human pathogen^11^; since then, our understanding of its potential as a pathogen has transformed significantly. It is now known to be associated with opportunistic nosocomial infections such as catheter-associated urinary tract infections, ventilator-associated pneumonia, and central line–associated bacteremia.^11^ *Serratia* may also cause severe infections following penetrating trauma, such as endocarditis following IV drug use,^12^ meningitis following spinal anesthesia,^13^ and spinal epidural abscess following acupuncture.^14^

*Serratia* skin and soft tissue infections are uncommon, and most cases occur in immunocompromised patients. Presentations include isolated plaques,^15^ bullous cellulitis,^16^ papillovesicular eruptions,^17^ abscesses,^18,19^ nodules,^20,21^ granulomatous lesions,^22,23^ and necrotizing cellulitis.^24^ Cases have been reported in the extremities of patients with uncontrolled diabetes^25^ and end-stage renal disease,^26^ as well as those who have undergone splenectomy^27^ and chemotherapy.^17^ Infections associated with immunocompromised state frequently disseminate and lead to life-threatening sepsis. In healthy, immunocompetent patients, *Serratia* skin and soft tissue infections are generally localized and associated with direct trauma or chronic vascular disease. Infections in patients with venous insufficiency have presented as nodules, purulent ulcers, and cellulitis.^18,20,21,28^

To better understand the risk factors and potential etiologies for infection in our patient, we conducted a review of reported cases of *Serratia marcescens* skin and soft tissue infections associated with inoculation injury or trauma (Table 1). Twenty-five individual patient cases were found. Skin and soft tissue manifestations included cellulitis (11 cases), necrotizing fasciitis (6 cases), cellulitis progressing to necrotizing fasciitis (3 cases), abscesses (2 cases), ulcers (2 cases), and inflamed nodule (1 case). Thirteen cases were reported in immunocompetent patients, 11 were in immunocompromised patients, and 1 did not specify. Two cases were in patients who were immunocompromised from chemotherapy.

**Table 1.** Cases of *Serratia marcescens* Skin and Soft Tissue Infections Following External Trauma.

Case	Patient Demographic	Preceding Traumatic Insult	Distribution	Immunocompromised	Skin/Soft Tissue Manifestation	Management	Outcome
Brenner and Lookingbill^45^	51-year-old male	Intravenous catheter placement	Thigh	No	Cellulitis	Nafcillin, gentamicin	Death from hepatic and renal failure 7 days after catheter placement
Bornstein et al^26^	37-year-old female	Hemodialysis, fistula-site needle penetration	Axilla, breast, thorax	Yes; end-stage renal disease	Cellulitis	Surgical debridement, amikacin, ciprofloxacin	Resolution
Bonner and Meharg^44^	60-year-old male	Muscle and nerve biopsy	Foot	No	Cellulitis	Cefoxitin	Resolution
Cooper et al^25^	69-year-old female	Ingrown toenail surgery	Foot	Yes; type 2 diabetes mellitus	Bullous cellulitis	Amputation	Resolution
Pereira et al^33^	21-year-old female	Finger amputations caused by steel door; reconstructive surgery; leech application	Third and fourth digits on hand	No	Cellulitis	Amputation of reconstructed fingertip, ciprofloxacin	Resolution
Hsieh and Babl^29^	8-year-old male	Iguana bite	Index finger	No	Cellulitis	Incision and drainage, ampicillin/sulbactam, gentamicin, amoxicillin/clavulanate	Resolution
Huang et al^43^	40-year-old male	Skin biopsy	Foot	Yes; prednisolone therapy for systemic lupus erythematosus	Necrotizing fasciitis	Surgical debridement, ceftazidime	Resolution
Curtis et al^34^	51-year-old male	Scraping legs on rocks while fishing in river	Leg	Yes; end-stage renal disease	Necrotizing fasciitis	Surgical debridement, vancomycin, ciprofloxacin, clindamycin, aztreonam	Resolution
Grim et al^16^	54-year-old male	Iguana bite	Posterior calf	No	Bullous cellulitis	Trimethoprim/sulfamethoxazole	Resolution
Grim et al^16^	25-year-old male	Iguana bite	Ankle	No	Bullous cellulitis	Trimethoprim/sulfamethoxazole	Resolution
Motsitsi^31^	37-year-old male	Human bite	Forearm	No	Necrotizing fasciitis	Surgical debridement	Death 2 days after admission
Park and Seo^47^	62-year-old female	Dermal filler injection	Upper face	No	Inflamed nodule	Trimethoprim/sulfamethoxazole	Resolution
Prelog et al^46^	15-year-old female	Venous access port implantation	Axilla	Yes; active chemotherapy for acute lymphoblastic leukemia	Necrotizing fasciitis	Surgical debridement	Resolution
Subramani et al^30^	50-year-old female	Snake bite	Hand	No	Bullous cellulitis and necrotizing fasciitis	Surgical debridement, ciprofloxacin, piperacillin/tazobactam	Resolution
Sharma et al^32^	11-month-old female	Insect bite	Upper chest	No	Abscess followed by ulceration	Ceftazidime, amikacin, amoxicillin/clavulanate	Resolution
Vano-Galvan et al^48^	57-year-old female	Minor trauma of unspecified origin	Thigh	Yes; chemotherapy for chronic lymphocytic leukemia, type 2 diabetes mellitus	Bullous cellulitis and necrotizing fasciitis	Linezolid, piperacillin/tazobactam	Death 2 hours after presentation
García et al^38^	32-year-old male	Tattoo	Elbow	No	Abscess	Incision and drainage, ertapenem, ciprofloxacin	Resolution
Lakhani et al^41^	51-year-old female	Bifemoral bypass and left distal femoral aneurysm repair surgeries	Abdomen and groin	Yes; type 2 diabetes mellitus	Necrotizing fasciitis	Ciprofloxacin	Resolution
Majumdar and Crum-Cianflone^42^	54-year-old female	Skin biopsy	Leg	Yes; end-stage renal disease, type 2 diabetes mellitus	Bullous cellulitis and necrotizing fasciitis	Surgical debridement, vancomycin, piperacillin/tazobactam, levofloxacin, clindamycin	Resolution
Roth^39^	54-year-old female	Transobturator sling surgery	Thigh	Not specified	Cellulitis	Surgical debridement, ciprofloxacin	Resolution
Hagiya et al^36^	64-year-old male	Untreated burn	Leg	Yes; liver cirrhosis	Necrotizing fasciitis	Penicillin G, meropenem, clindamycin	Death from septic shock 25 hours after presentation
Kyvernitakis et al^40^	71-year-old female	Mastectomy and sentinel node biopsy	Breast	No	Cellulitis with underlying seroma	Aspiration, ciprofloxacin	Resolution
Veraldi and Nazzaro^37^	75-year-old male	Scratch from bush	Leg	No	Ulcer	Ceftriaxone	Resolution
Veraldi and Nazzaro^37^	75-year-old female	Scratch from rose thorn	Leg	Yes; type 1 diabetes mellitus	Ulcer	Levofloxacin	Resolution
Marin et al^35^	50-year-old male	Dropping of piece of drywall to affected area	Foot	Yes; type 2 diabetes mellitus	Bullous cellulitis, liquefactive necrosis, necrotic eschar	Incision and drainage; discharged against medical advice before antibiotic treatment	Lost to follow-up

Five of the reports were associated with bite trauma, including 3 iguana bites,^16,29^ 1 snake bite,^30^ 1 human bite,^31^ and 1 insect bite.^32^ One case followed exposure to leeches.^33^ Six of the cases had preceding accidental trauma or burns.^33-35^ Three of the cases involved outdoor contact, including scrapes by rocks, bushes,^36^ and a rose thorn.^37^ One case of an abscess caused by a tattoo was reported.^38^ There are 12 reports of occurrences subsequent to iatrogenic trauma, including 5 that occurred after surgery.^25,33,39-41^ Other iatrogenic-associated cases followed skin or muscle biopsies,^42-44^ IV catheter placement,^45,46^ arteriovenous fistula,^26^ and dermal filler injection.^47^ One patient was described to have “minor trauma” of unspecified origin.^48^ Most of these infections were managed successfully with antibiotics or surgical debridement, though 4 were fatal.

Based on our review, this may be the first report of *Serratia marcescens* skin infection associated with traumatic break in the skin caused by a dog scratch. The source of *Serratia* in our patient is unclear, but it may have been introduced into the wound by the dog scratch, while cleaning the wound with contaminated isopropanol, or while taking a shower. Risk factors included her profoundly neutropenic state on presentation and her exposure to prophylactic antibiotics during and after chemotherapy. Her chest port, which was located on the same side as her affected arm, was also considered a potential nidus for infection leading to secondary cellulitis. This is less likely given the absence of *Serratia* bacteremia and negative culture of port following removal.

## Conclusion

We report a case of *Serratia marcescens* cellulitis following a dog scratch in a neutropenic patient. This case highlights the need for clinicians to consider unusual pathogens based on exposure history in cases of treatment-resistant soft tissue infections in immunocompromised patients. It also emphasizes the importance of obtaining cultures from drainable lesions or tissue to establish a microbiologic diagnosis and allow for targeted therapy.
